# Assessment of body Schema distortions in early-onset schizophrenia

**DOI:** 10.1016/j.scog.2024.100320

**Published:** 2024-06-28

**Authors:** Marine Fiorentino, Arnaud Carré, Laura Vandemeulebroucke, Morgane Metral

**Affiliations:** a Univ. Grenoble Alpes, LIP/PC2S, 38000 Grenoble, France; b Dispositif Ressources Autismes, Centre Hospitalier Spécialisé de la Savoie, 73000 Bassens, France; c Unité d'Accueil Pédiatrique de l'Enfant en Danger, Centre Hospitalier Metropole Savoie, Chambéry, France

**Keywords:** Early-onset schizophrenia, Assessment, Body schema, Motor imagery, Early diagnosis

## Abstract

Distorted body representations play a major role in the onset and maintenance of Schizophrenia. However, these distortions are difficult to assess because explicit assessments can provoke intense fears about the body and require a good insight. We proposed an implicit motor imagery task to a 14-year-old girl with Early-Onset Schizophrenia. The test consisted of presenting different openings varying in width. For each aperture, the young girl has to say if she could pass through without turning her shoulders. A critical aperture is determined as the first aperture for which she considered she could no longer pass, compared to her shoulders' width. The girl perceived herself as 51 % wider than she was, indicating a significant oversized body schema. The implicit assessments of body schema generate less anxiety and does not require a great level of insight; moreover, those are promising tools for early detection of disease in prodromal phases of Schizophrenia and assistance with differential diagnosis.

The body schema is a sensory-motor, automatic, and constantly readjusted representation of the global and/or segmental position of the body and its movements. This representation involves different sensory systems, their coordination, and their integration (vision, tact, proprioception, interoception**)** and may be solicited without consciousness (de Vignemont, 2010; de Vignemont et al., 2021; Pireyre, 2021). Each individual refers automatically to this representation, as it is involved in guiding actions adjusted to one's own morphology.

In schizophrenia, the body schema is altered, with the body's boundaries always depicted as *“blurred and permeable,”* there being a disturbed sense of body ownership and body self-consciousness (Klaver and Dijkerman, 2016; Mirucka, 2019; Szczotka and Majchrowicz, 2018; Tordjman et al., 2019), preventing the person from correctly differentiating their body from the environment. These body schema distortions are linked to sensory processing problems (Fletcher and Frith, 2009; Ford et al., 2012; Klaver and Dijkerman, 2016; Yao and Thakkar, 2022) and have consequences for motor skills (Lafargue and Franck, 2009).

Assessing body representation disorders, in particular body schema disorders, is complex, even more so in early-onset schizophrenia, when the individual has poor insight and major anxiety about the body. Indeed, disturbances in body representation are often assessed by asking the individual to draw a stick figure or use modeling clay, or by evoking bodily sensations (Boutinaud, 2017). Other tools such as questionnaires also exist. For example, the 84-item Body Distortion Questionnaire investigates various body distortions (e.g., “My body seems small to me”; “My body seems unprotected to me”). The overall distortion score is based on seven categories: size, smallness, boundaries, blockage, skin, dirtiness, depersonalization (Sudres, 2004). Another questionnaire on bodily experience uses 52 items to investigate negative feelings about the body, body perceptions, and the importance of the body's interior (Sudres, 2004). Despite interest in these measures, they are often unusable in early-onset schizophrenia, first, because the fears provoked by the questions make them difficult to use, and second, because these questionnaires require a good level of insight, which is often missing in individuals with schizophrenia.

We were asked to assess a young woman's representation of her body. The 14-year-old female patient was diagnosed with early-onset schizophrenia (American Psychiatric Association, 2013) and has difficulties with body perception. Indeed, she reported having major problems with body awareness. The young girl appears to have severe difficulty with her own body daily, *“as she does not perceive its limits or dimensions, and consequently has difficulty situating herself in space, as a person differentiated from the environment,”* according to her medical record. This relationship with the body was a source of immense daily anxiety. As expected, the use of explicit tools to evaluate her body representation was not possible because of this massive anxiety and poor insight. Our objective was to assess her body representation distortions by using an implicit task that would enable us to quantify her body schema distortion without causing anxiety.

The tool we were able to use to assess the body schema was inspired by Brownell et al. (2007). They presented one door of the right size for a child to walk through, whereas the second door was far too narrow for the child to even put his or her head through. The authors made a binary assessment of a major alteration in the child's body schema. We proposed to the patient the use of a more complete motor imagery aperture task, one that is already being used to assess the body schema, for example, in those with anorexia nervosa (Guardia et al., 2010, Guardia et al., 2012; Metral et al., 2014). We adapted this task to the patient's clinical environment, age, and cognitive abilities. The test consisted of presenting an opening whose width varied from 30 to 90 cm in increments of 10 cm (Fig. 1). The young girl stood 5 m from the opening presented and had to indicate for each opening width whether she could pass through it by imagining herself walking through without turning her shoulders. The width at which the patient thought she could no longer pass through was the critical opening. The critical opening (in millimeters) was then divided by the width of the girl's shoulders (in millimeters). This gives the percentage of body schema distortion. In healthy subjects, there is a 15 % margin of error in the ratio between the critical opening width and the width of the shoulders (Guardia et al., 2010; Metral et al., 2014, Metral et al., 2020; Warren and Whang, 1987). The results showed that the patient had distorted body dimensions, perceiving herself to be 51 % wider than she was compared with what is observed in different control groups during the same task (Guardia et al., 2010; Metral et al., 2014; Warren and Whang, 1987). This case study highlights that in early-onset schizophrenia, despite the patient having intense phobias, the body schema may still be assessed by using an implicit task, such as a motor imagery task.

**Fig. 1:**
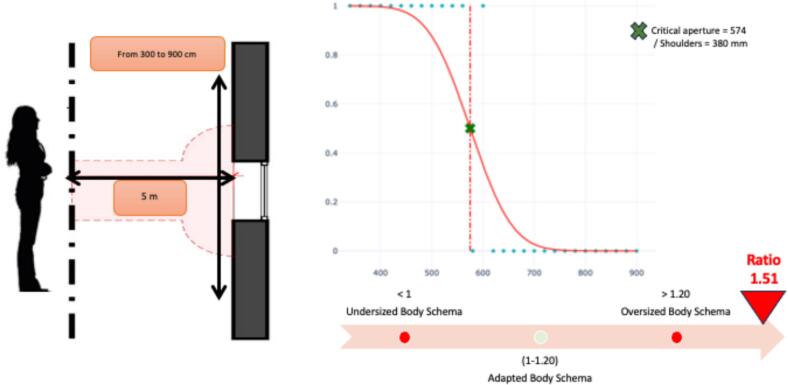
**Left Panel**: Illustration of the body schema implicit assessment in a motor imagery task in which an aperture was used that varied from 30 to 90 cm by increments of 10 cm. **Right Panel**: Graph showing the girl's responses as a function of the width of the opening presented (in millimeters). A theoretical critical opening (green cross) is automatically calculated and represents the opening at which the girl thinks her body no longer fits through the opening. The ratio of opening width to shoulder width is calculated. A ratio of 1.51 is well above the norm and indicates an oversized body schema.

Normally, to assess the body schema, a real motor task would be necessary to evaluate movements in the environment and to check whether they are adapted to the individual's morphology. This approach requires the use of cameras and movement processing software (Baumann et al., 2022; Beckmann et al., 2021; Keizer et al., 2013; Metral et al., 2014). The use of a motor imagery task, rather than a real motor task, is still valid for assessing the body schema, since real motricity and motor imagery share similar neural networks and both rely on the body schema (Hétu et al., 2013). Moreover, the use of a motor imagery task makes it easier to assess the body schema, as it requires fewer resources than a real motor task does. This imagery task is also often used in research to determine the impact of experimental manipulation of the body schema (Beaudoin et al., 2020; Dupraz et al., 2022), as well as body schema distortions in anorexia nervosa (Guardia et al., 2010, Guardia et al., 2012; Metral et al., 2014).

Even though the motor imagery task we proposed was easier to use than a real motor task, we regret that in clinical practice, the body schema is not often assessed, as it requires more complex logistics than occurs with questionnaires or drawings. However, in early-onset schizophrenia, the fears and difficulties associated with an explicit assessment of one's body perception cannot be compatible with an explicit assessment of the body (phobias, poor insight). In addition, to draw any conclusions about distortions of body perception, explicit evaluations about emotional and attitudinal components of the body are not suitable compared with an evaluation of body schema. From this perspective, we have also developed an even easier method than a motor imagery task to assess the body schema, in the form of an e-health platform with two tasks: a virtual reality motor task (MOVE) and a tactile estimation task (SKIN) to assess the motor and tactile dimensions of the body schema, respectively. All of the tools are connected to a web application,^1^ which determines whether the participant's scores are within the norm. The aim is to enable medical practitioners to assess body schema distortions in all body representation disorders (chronic pain, anorexia nervosa, autism spectrum, etc.).

In parallel, the results of studies about schizophrenia highlight the importance of using standardized tools for diagnosis in the pediatric population. For example, early-onset schizophrenia might be underdiagnosed in children and adolescents with neurodevelopmental disorders and subnormal cognitive functioning (Dor-Nedonsel et al., 2020). In the context of screening for those disorders in children, assessment of the body schema seems to be even more crucial, as anomalies in embodiment appear during the prodromal phases of schizophrenia (Nelson et al., 2012). Moreover, body schema disturbances in schizophrenia have different signatures than those observed in other differential diagnoses (e.g., autism spectrum) (Asada et al., 2016; Ropar et al., 2018).

We recommend the systematic use of implicit and ecological tasks to evaluate body schema in early-onset schizophrenia because they seem particularly appropriate in limiting the fears and difficulties associated with an explicit assessment of one's body perception (e.g., questionnaires, drawings). In addition, implicit tasks ensure that the evaluation is related to distortions of body self-consciousness and the perception of body limits (i.e., body schema), rather than emotional and attitudinal components of body image. Finally, these tools are promising for early detection of disease in the prodromal phases of schizophrenia and may assist with the differential diagnosis in the pediatric population.

## Ethics approval statement

The study was conducted in accordance with the Declaration of Helsinki, and the protocol was approved by the Ethics Committee of routine clinical practice protocol for patient care.

## Funding statement

The conduct of the research and/or the preparation of the article did not require any specific funding. However, the tools used to assess the body schema, as evoked in the discussion section, have been funded by the Foundation of ‘Université Savoie Mont Blanc’ and the ‘SATT Linksium’.

## CRediT authorship contribution statement

**Marine Fiorentino:** Writing – review & editing, Validation, Project administration, Conceptualization. **Arnaud Carré:** Writing – review & editing. **Laura Vandemeulebroucke:** Writing – review & editing, Methodology, Investigation. **Morgane Metral:** Writing – original draft, Project administration, Methodology, Data curation, Conceptualization.

## Declaration of competing interest

The authors declare no conflicts of interest.

## Data Availability

The data that support the findings of this letter are available on request from the corresponding author. The data are not publicly available due to privacy or ethical restrictions.

## Footnotes

1 https://servo.psycho-usmb.fr/
